# Carbapenem-Resistant *Enterobacteriaceae* in Urinary Tract Infections: From Biological Insights to Emerging Therapeutic Alternatives

**DOI:** 10.3390/medicina60020214

**Published:** 2024-01-26

**Authors:** Eugenio Bologna, Leslie Claire Licari, Celeste Manfredi, Francesco Ditonno, Luigi Cirillo, Giovanni Maria Fusco, Marco Abate, Francesco Passaro, Ernesto Di Mauro, Felice Crocetto, Savio Domenico Pandolfo, Achille Aveta, Simone Cilio, Isabella Di Filippo, Biagio Barone, Antonio Franco, Davide Arcaniolo, Roberto La Rocca, Biagio Pinchera, Luigi Napolitano

**Affiliations:** 1 Unit of Urology, Department of Maternal-Child and Urological Sciences, Policlinico Umberto I Hospital, “Sapienza” University, 00161 Rome, Italy; eugenio.bologna@uniroma1.it (E.B.); leslieclaire.licari@uniroma1.it (L.C.L.); 2 Unit of Urology, Department of Woman, Child and General and Specialized Surgery, University of Campania “Luigi Vanvitelli”, 80131 Naples, Italy; davide.arcaniolo@gmail.com; 3 Department of Urology, University of Verona, Azienda Ospedaliera Universitaria Integrata, 37100 Verona, Italy; francesco.ditonno@icloud.com; 4 Department of Neurosciences, Science of Reproduction and Odontostomatology, University of Naples Federico II, 80131 Naples, Italy; cirilloluigi22@gmail.com (L.C.); giom.fusco@gmail.com (G.M.F.); marcoabate5@gmail.com (M.A.); francescopassaro1996@gmail.com (F.P.); ernestodm9@gmail.com (E.D.M.); felice.crocetto@unina.it (F.C.); pandolfosavio@gmail.com (S.D.P.); achille-aveta@hotmail.it (A.A.); simocilio.av@gmail.com (S.C.); robertolarocca87@gmail.com (R.L.R.); nluigi89@libero.it (L.N.); 5 Department of Urology, University of L’Aquila, 67010 L’Aquila, Italy; 6 Department of Clinical Medicine and Surgery, Section of Infectious Diseases, University of Naples “Federico II”, 80131 Naples, Italy; isadifi93@gmail.com (I.D.F.); biapin89@virgilio.it (B.P.); 7 Division of Urology, Department of Surgical Sciences, AORN Sant’Anna e San Sebastiano, 81100 Caserta, Italy; biagio.barone@aorncaserta.it; 8 Department of Urology, Sant’Andrea Hospital, “Sapienza” University, 00189 Rome, Italy; anto.franco@hotmail.it

**Keywords:** antibiotic resistance, carbapenem resistant, CRE, MDR, UTI

## Abstract

Urinary tract infections (UTIs) are the second most frequent type of infection observed in clinical practice. Gram-negative *Enterobacteriaceae* are common pathogens in UTIs. Excessive antibiotic use in humans and animals, poor infection control, and increased global travel have accelerated the spread of multidrug-resistant strains (MDR). Carbapenem antibiotics are commonly considered the last line of defense against MDR Gram-negative bacteria; however, their efficacy is now threatened by the increasing prevalence of carbapenem-resistant *Enterobacteriaceae* (CRE). This comprehensive review aims to explore the biological mechanisms underlying carbapenem resistance and to present a focus on therapeutic alternatives currently available for complicated UTIs (cUTIs). A comprehensive bibliographic search was conducted on the PubMed/MEDLINE, Scopus, and Web of Science databases in December 2023. The best evidence on the topic was selected, described, and discussed. Analyzed with particular interest were the clinical trials pivotal to the introduction of new pharmacological treatments in the management of complicated cUTIs. Additional suitable articles were collected by manually cross-referencing the bibliography of previously selected papers. This overview provides a current and comprehensive examination of the treatment options available for CRE infections, offering a valuable resource for understanding this constantly evolving public health challenge.

## 1. Introduction

Urinary tract infections (UTIs) are among the most common infectious diseases encountered in medical settings, affecting an estimated 150 million individuals annually worldwide. This high prevalence ranks UTIs as the second most frequent type of infection observed in clinical practice [1].

Clinical presentations range from benign, uncomplicated infections to complicated UTIs (cUTIs), pyelonephritis and severe urosepsis [2]. In addition to the higher risk of severe outcomes, cUTIs are characterized by a higher risk of recurrence or chronicity than uncomplicated UTIs, making its treatment an ever-evolving challenge.

Gram-negative *Enterobacteriaceae* are common pathogens in UTIs. These pathogens initially posed a threat to the public health due to their ability to become resistant to antibiotics by producing extended-spectrum ß-lactamases (ESBLs) [3]. Moreover, excessive antibiotic use in humans and animals, poor infection control, and increased global travel have accelerated the spread of multidrug-resistant (MDR) strains [4,5]. From this point of view, cUTIs and pyelonephritis are associated with high antimicrobial resistance rates among causative pathogens than simple UTIs.

The phenomenon of bacterial resistance escalated rapidly as the prevalence of ESBL Gram-negative bacteria increased, leading to a reliance on carbapenems—a broad-spectrum antimicrobial agent—as first-line empirical treatments [6,7,8]. This class of antibiotics are frequently considered the best defense against MDR Gram-negative bacteria, but their efficacy is now threatened by the growing prevalence of carbapenemase-producing pathogens [9]. In fact, initially embraced as the treatment of choice against ESBL-producing *Enterobacteriaceae*, carbapenems inadvertently fostered the advent of carbapenem-resistant *Enterobacteriaceae* (CRE). The Center for Disease Control and Prevention (CDC) defines CRE as *Enterobacteriaceae* that exhibit resistance to carbapenem antibiotics or are confirmed producers of carbapenemase enzymes [10]. The persistence and propagation of CRE, despite a high concentration of carbapenems, significantly hinders the efficacy of existing treatments for infectious diseases, emphasizing the complex repercussions of antibiotic use in clinical settings.

Pyelonephritis and cUTIs have emerged as infection models for the study of novel antibiotics, including extensive investigation and clinical trials of new therapies against Gram-negative bacteria.

This review aims to elucidate the biological underpinnings of carbapenem resistance in cUTIs and to critically evaluate the therapeutic alternatives currently available for this pressing public health issue.

## 2. Materials and Methods

We designed a narrative review. A comprehensive bibliographic search was conducted in December 2023 using PubMed/MEDLINE, Scopus, and Web of Science databases.

Prospective and retrospective study were included. Special attention was given to clinical trials pivotal to the introduction of new pharmacological treatments in the management of complicated cUTIs. Relevant preclinical studies were also included. Additional articles were identified by manually cross-referencing the bibliographies of selected papers. Selection of papers was based on the authors’ experience.

Data extraction was performed by two independent reviewers (E.B and L.C.L.) using a standardized form. Disagreements were resolved through discussion and a third senior author (C.M.) was consulted for consensus when necessary.

Data were reported in the main text and tables as in the original articles without applying specific statistical tests.

## 3. Epidemiology

In the past decade, there has been a worrying rise in the global spread of CRE infections [11]. These infections are a significant healthcare concern due to their association with high morbidity and mortality rates, particularly among vulnerable patient populations [12]. The incidence of CRE infections varies, ranging from 0.46 per 10,000 patient-days to 4.17 per 10,000 patient-days [13].

In Europe, data from Italy and Greece show that CRE pathogens contribute to an estimated 30,000 deaths annually [14,15]. The United States faces around 9000 healthcare-associated CRE infections each year, leading to approximately 600 deaths, which equates to a 6.6% mortality rate [16,17,18]. The situation is even more dire in developing regions such as Asia, Africa, and South America, where morbidity and mortality rates from MDR infections are higher [19]. In China, the resistance to imipenem among *E. coli* and *K. pneumoniae* increased from approximately 0.7% in 2004–2005 to 2.7% by 2010 [20].

The problem of the increasing incidence of CRE was also reported for UTIs all over the world [21]. Most of the largest studies in this field have been performed in Asian countries, particularly Sri Lanka, India, and China, where CRE accounts for about 11% of UTIs [22,23].

## 4. Mechanisms of Drug Resistance

**Figure 1:**
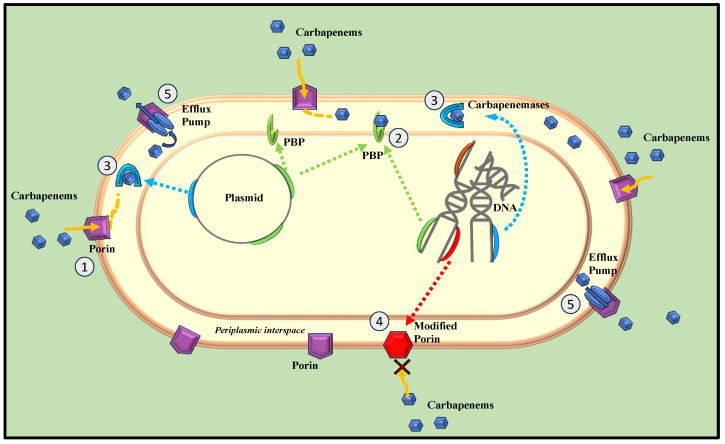
Principal mechanisms of carbapenem-resistant Enterobacteriaceae. The uptake of carbapenems through the bacterium’s outer membrane is facilitated by hydrophilic channels formed by porins (1). Once inside, these antibiotics irreversibly bind to penicillin-binding proteins (PBPs) in the periplasmic space, leading to the inhibition of peptidoglycan synthesis (2). The primary mechanisms of carbapenem resistance include enzymatic inactivation by chromosome- and/or plasmid-encoded hydrolytic enzymes (3), reduced permeability of the outer membrane due to altered porin production (4), and the efflux of antibiotics from the bacterium via efflux pumps (5).

Over the past decade, *Enterobacteriaceae* have developed resistance to carbapenems primarily through three major mechanisms: synthesis of carbapenemase enzymes, efflux pumps, and membrane permeability changes due to porin mutations [24] (Figure 1). Generally, CRE are divided into two main subgroups: carbapenemase-producing CRE (CP-CRE) and non-carbapenemase-producing CRE (non-CP-CRE) [17].

However, these mechanisms generally appear paired among themselves or with carbapenemase production. In fact, while carbapenemases specifically target carbapenems and other ß-lactam antibiotics, efflux pump expression or porin changes are associated with MDR [24].

### 4.1. Carbapenemase Enzymes

Carbapenemases are the most versatile β-lactamase family [25]. Despite their designation as “carbapenemases”, these enzymes can hydrolyze almost all β-lactams and are often resistant to all commercially available β-lactamase inhibitors. Some researchers advocate for ‘carbapenem-hydrolyzing enzymes’ as a more accurate term to describe these enzymes, noting that carbapenems are only a fraction of their extensive substrate spectrum [26].

The synthesis of this class of enzymes constitutes one of the most significant mechanisms contributing to carbapenem resistance in *Enterobacteriaceae* [27]. Since their discovery, numerous classifications have been proposed. Currently, they are divided into three subclasses based on a combination of structural features, zinc affinities, and hydrolysis profiles [28]. Carbapenemases are classified within Ambler classes A, B, and D. Molecular classes A and D include β-lactamases that have serine at their active sites, whereas class B β-lactamases are zinc-dependent metalloenzymes.

Class A carbapenemases notably include the NMC/IMI (not metalloenzyme carbapenemase/imipenem-hydrolyzing β-lactamase), SME (Serratia marcescens enzyme), KPC (Klebsiella pneumoniae carbapenemase), and GES (Guiana extended spectrum) enzymes [29].

Most of these enzymes require a serine residue at the active site, specifically at position 70 based on Ambler’s classification, for their hydrolytic action [30]. They can degrade a wide range of β-lactams, including carbapenems, cephalosporins, penicillins, and aztreonam, and are inhibited by clavulanate and tazobactam, classifying them within the group 2f β-lactamases [29].

Chromosomally encoded class A carbapenemases, such as SME, NMC, and IMI—initially detected in *Enterobacter cloacae*, *Serratia marcescens*, and *Klebsiella* spp. [31]—demonstrate a broad hydrolysis range, encompassing penicillins, early cephalosporins, aztreonam, and carbapenems.

Two distinct properties differentiate KPC carbapenemases from other enzymes in this functional group. Firstly, KPC enzymes are encoded on transmissible plasmids, and secondly, they exhibit a substrate hydrolysis profile that extends to aminothiazoleoxime cephalosporins like cefotaxime [32].

Specifically, KPC carbapenemases are capable of hydrolyzing all classes of β-lactams, showing the highest efficiency with compounds such as nitrocefin, cephalothin, cephaloridine, benzylpenicillin, ampicillin, and piperacillin. Imipenem, meropenem, cefotaxime, and aztreonam are hydrolyzed with less efficiency—approximately tenfold lower than penicillins and early cephalosporins. However, they can still perform weak but detectable hydrolysis of cefoxitin and ceftazidime, which contributes to the KPC enzymes’ extensive hydrolysis range encompassing most β-lactam antibiotics.

The genes for the GES enzyme family are located within integrons on plasmids. Initially, due to their wide hydrolytic range encompassing penicillins and extended-spectrum cephalosporins, these enzymes were categorized as extended-spectrum β-lactamases (ESBLs) [33]. Characterized by only two amino acid changes, they retain class A β-lactamase active site motifs, with cysteine residues at the Ambler positions 69 and 238.

Class B β-lactamases are known for their carbapenem hydrolysis capability and their resistance to commercially available β-lactamase inhibitors; however, they are vulnerable to metal ion chelators like EDTA. These enzymes have a broad substrate range, hydrolyzing carbapenems, cephalosporins, and penicillins, but not aztreonam. The hydrolysis process relies on the interaction with zinc ions at the enzyme’s active site. In contrast to class A, class B requires Zn^2+^ for effective hydrolysis and is not inhibited by clavulanic acid or tazobactam.

Class D β-lactamases encompass the OXA group, which are oxacillin-hydrolyzing β-lactamases. This group was one of the most widespread plasmid-encoded β-lactamases, especially among *Enterobacteriaceae* and *P. aeruginosa*, in the late 1970s and early 1980s [34,35]. The OXA β-lactamases utilize a catalytic mechanism similar to other serine carbapenemases, forming a covalent acyl–enzyme intermediate with the catalytic serine residue, followed by deacylation that results in the inactive hydrolyzed antibiotic at the C-N bond of the β-lactam ring [36]. OXA carbapenemases are active against penicillins, certain cephalosporins, and carbapenems.

### 4.2. Alteration of Membrane Permeability

A pivotal mechanism by which non-CP-CRE evade the efficacy of carbapenem antibiotics is through the alteration of their membrane permeability [37,38]. This adaptive change is primarily facilitated by mutations in membrane porin proteins. These porins function as gateways, forming channels through the bacterial outer membrane, thus permitting the antibiotics access to bacterial targets.

The genetic basis for these modifications can be traced to mutations within the gene sequences that encode for these porins. Deletions, insertions, or single-nucleotide substitutions within these genes can lead to a phenotypic expression that results in either a reduction in the size of porin channels or a change in the electrostatic charge of the porins, both of which critically hinder the uptake of carbapenem antibiotics.

Significant resistance in CRE is associated with the dysfunction of critical outer membrane proteins, particularly OmpK36 and OmpK35. The impairment of these proteins restricts the entry of charged molecules, including antibiotics, which is crucial for the bacteria’s defense against these drugs.

Hao et al. reported that mutations in both OmpK36 and OmpK35 lead to higher carbapenem resistance compared to mutations in OmpK36 alone [39].

Hamzaoui et al. similarly highlights that in CRE *K. pneumoniae* isolates there is a loss of both the major porins or mutations within the genes regulating the porin system. The most prevalent mutations involve the transcription factor OmpR, which moderates the expression of outer membrane proteins [40]. Additionally, Kong et al. discovered a novel mutation within the N-terminal phosphorylation domain of OmpR—G63S—that impacts membrane sensor kinases [41]. Failure to phosphorylate OmpR results in a deficit in porin transcription with a consequent change in membrane permeability.

Mutations in micC and micF genes, which control porin gene expression through antisense RNAs, are also implicated in porin loss. Hao et al. show that an overexpression of these genes can lead to a marked decrease in major porin production [39].

While a reduction in membrane porins alone only decreases antibiotics susceptibility, when coupled with β-lactamase production, it contributes to full resistance. This is due to a synergistic effect where the decreased antibiotic uptake in the periplasm and cytosol enhances the efficacy of β-lactamases [42].

### 4.3. Overexpression of Efflux Pumps

Efflux pumps are critical membrane proteins that actively transport substrates, including antibiotics, from the interior of a bacterial cell to the external environment. In the context of CRE, both overexpression of efflux pumps and mutations of these proteins —that makes them more efficient at exporting substrates—significantly correlates with a resistant bacterial phenotype to antibiotics [43].

A well-known multidrug resistance efflux pump system is AcrAB-TolC, part of the Resistance–Nodulation–Division (RND) superfamily. This system comprises three components: AcrA, a periplasmic membrane fusion protein; AcrB, an inner membrane transporter; and TolC, an outer membrane protein [44,45,46,47,48]. This system and/or TolC alone have been suggested as a potential target for efflux inhibitors. An efflux inhibitor can disrupt the proton motive force across the bacterial membrane, leading to depolarization and loss of the electrochemical concentration gradient necessary for pump function. However, Saw et al. demonstrated that the inhibition of the efflux pump system or loss of a component such as TolC increased the resistance of bacteria to some antibiotics which use outer membrane porins as entry routes into the bacterial cell [47]. The authors suggested that this was likely due to changes in porin expression complex, highlighting the difficulties in identifying an ideal drug target in such a complex and variable microenvironment. Furthermore, the expression of the AcrAB-TolC efflux system is regulated by global regulators such as the AraC-type antibiotic resistance regulator A (RarA) [49]. Chetri et al. demonstrated a strong correlation between RarA expression and ertapenem concentration, resulting in upregulation of AcrAB expression and reduced susceptibility to carbapenems in *E. coli* clinical isolate [46]. Moreover, mutations in other transporters of the RND superfamily, such as AcrD, can act as a compensatory mechanism for the loss of AcrB. These mutations may enhance the export of carbapenems from the periplasm, contributing to the bacteria’s ability to resist carbapenems [44].

From this perspective, it becomes evident that identifying targets to counteract pharmacological resistance presents a complex challenge and remains a topic of ongoing and evolving research.

## 5. Current and Future Treatment Options for Urinary Infections Caused by CRE

**Figure 2:**
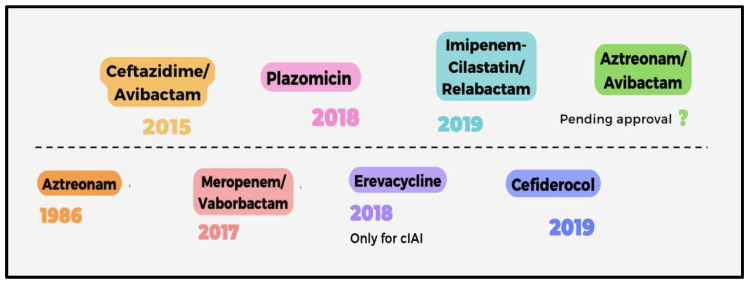
Timeline regarding the US Food and Drug Administration (FDA) approval of the antibiotic agents discussed in this review. Eravacycline was not approved for cUTI. Aztreonam–Avibactam is still under investigation for cUTIs. cIAI = complicated intra-abdominal infection.

A summary of the selected randomized controlled trials (RCTs) pertinent to current treatment modalities for UTIs are detailed in Table 1. Table 2 delineates the principal characteristics of the antibiotics under discussion, encapsulating their mechanisms of action and indications against resistant strains. A timeline describing the therapies approved by the FDA is depicted in Figure 2.

### 5.1. Ceftazidime/Avibactam

Ceftazidime is a third-generation broad-spectrum cephalosporin antibiotic. It works as a bactericide by binding to and inhibiting penicillin-binding proteins (PBPs), which are crucial for synthesizing bacterial cell walls through the synthesis and remodeling of peptidoglycan. This drug is effective against a wide range of Gram-negative bacteria, including strains of *N. gonorrhoeae* that produce penicillinase and various members of the *Enterobacteriaceae* family [63]. Among all cephalosporins, ceftazidime shows the highest activity against *Pseudomonas aeruginosa*.

Avibactam, on the other hand, is a β-lactamase inhibitor that does not possess inherent antibiotic activity. It is used to enhance the effectiveness of ceftazidime by protecting it from degradation and bacterial resistance mechanisms. Avibactam primarily targets class A and class C β-lactamases, with a lesser effect on class D enzymes. However, it does not inhibit metallo-β-lactamases (Class B β-lactamases) found in anaerobic bacteria and some species of *Pseudomonas* spp. [64].

When evaluating the efficacy of the combination therapy ceftazidime–avibactam (CAZ-AVI) against carbapenemase-producing *Enterobacteriaceae* isolates, it exhibited similar levels of sensitivity compared to colistin and tigecycline, with sensitivity rates of 73%, 77%, and 78.1%, respectively. However, when isolates producing metallo-β-lactamase (MBL) were removed from the analysis, the sensitivity of CAZ-AVI increased significantly, showing an effectiveness of 95.9% against the remaining carbapenemase-producing *Enterobacteriaceae* [65].

CAZ-AVI received approval from the U.S. FDA in 2015 for use in adults[66]. This approval covered its use for the treatment of cUTIs and complicated intra-abdominal infections (cIAIs) in combination with metronidazole.

Clinical trials have supported the safety and effectiveness of CAZ-AVI for these indications. Specifically, a prospective, phase II, randomized, investigator-blinded study by Vazquez et al. compared the efficacy and safety of CAZ-AVI with imipenem–cilastatin in hospitalized adults with severe cUTIs [50]. The study found a favorable microbiological response in 70.4% of patients treated with CAZ-AVI and 71.4% of those receiving imipenem–cilastatin. In patients with ceftazidime-resistant pathogens, an 85.7% response rate was observed in those treated with CAZ-AVI. Comparable outcomes were achieved in the treatment of cIAIs when comparing CAZ-AVI plus metronidazole with meropenem [67]. The antimicrobial effectiveness and safety of CAZ-AVI against contemporary pathogens causing cUTIs and cIAIs was subsequently confirmed in the pediatric population as well [68,69].

The RECAPTURE trial, acknowledging the urgent need to lessen reliance on carbapenems, assessed the efficacy and safety of CAZ-AVI versus doripenem in treating patients with cUTIs, including acute pyelonephritis [51]. Hospitalized adults were randomized in a 1:1 ratio to receive either CAZ-AVI at a dosage of 2000 mg/500 mg or doripenem at 500 mg, both administered every 8 h. The trial established the non*inferiority of CAZ-AVI compared to doripenem based on two co-primary endpoints: patient-reported symptomatic resolution and a combination of symptomatic resolution with microbiological eradication at the test-of-cure (TOC) visit. This trial demonstrates the high efficacy and cost-effectiveness of CAZ-AVI for the empirical treatment of cUTIs [70].

The recent EZTEAM study has provided Insights into the usage patterns of CAZ-AVI, including its indications and the antibiotics used in combination with it, as well as its effectiveness and safety in actual clinical settings [71]. The primary sources of infection identified in the study were intra-abdominal (17.4%), urinary (20.0%), and respiratory (22.1%). CAZ-AVI was primarily employed as a second-line treatment for Gram-negative infections and was often used alongside other antibiotics. The findings from this real-world study indicate that CAZ-AVI should be considered as a treatment option for MDR bacterial infections.

### 5.2. Meropenem/Vaborbactam

Meropenem, a carbapenem antibacterial agent, is resistant to hydrolysis by most β-lactamases produced by both Gram-negative and Gram-positive bacteria, including penicillinases and cephalosporinases. It achieves its bactericidal effect by binding to PBPs and ultimately resulting in cell death [72]. Vaborbactam is a broad-spectrum, non-suicidal β-lactamase inhibitor tailored to effectively inhibit class A serine carbapenemases, including KPC, NMC-A, and SME-2, along with other class A β-lactamases and class C β-lactamases. However, it does not inhibit class B carbapenemases, such as NDM, or class D carbapenemases [73,74].

The TANGO I study, a randomized, double-blind, multinational phase 3 trial, assessed the effectiveness of meropenem/vaborbactam (M/V) compared to piperacillin/tazobactam (P/T) in adults with cUTIs [52]. Specifically, the study evaluated the efficacy of M/V (2 g/2 g IV over 3 h every 8 h) against P/T (4 g/0.5 g IV over 30 min every 8 h). The FDA’s primary endpoint was the combination of clinical cure and microbiological eradication at the end of the intravenous treatment, while for the EMA, the primary endpoint was microbial eradication at the TOC visit. Out of 545 patients who were randomized and received at least one dose of the antibiotics (272 received M/V, 273 received P/T), the overall response at the TOC time-point (days 15–19) decreased in both groups—compared to the earlier assessment—but remained higher in the M/V group (74.5% vs. 70.3%). M/V resistance was noted in a single case of *Enterobacterales* (*K. pneumoniae* carrying OXA-48) and in 43% of *P. aeruginosa* isolates.

In 2017, the TANGO II trial, a subsequent randomized open-label controlled study, concluded its evaluation of the efficacy and safety of M/V monotherapy [75]^.^ It included patients with various CRE infections, such as UTIs, hospital-acquired and ventilator-associated pneumonia (HAP/VAP), cIAIs, and bloodstream infections (BSIs). Patients were randomized in a 2:1 ratio to receive M/V or the best available therapy (BAT), which was determined by an unblinded investigator and could include polymixins, carbapenems, aminoglycosides, or tigecycline, alone or in combination, as well as ceftazidime/avibactam monotherapy.

In cases of microbiologically confirmed CRE infection, M/V was linked to higher rates of clinical cure than BAT at both the end-of-treatment [65.6% vs. 33.3%, *p* = 0.03] and TOC [59.4% vs. 26.7%, *p* = 0.02] time-points. Moreover, microbiologic cures at the end of treatment were more frequent in the M/V group compared to BAT [65.6% vs. 40.0%; *p* = 0.09]. Notably, among patients with cUTIs, the rates of overall success at the end of treatment were higher for those who received M/V than for those who received BAT [75.0% vs. 50.0%].

Interestingly, a retrospective study conducted in 2020 examined the comparative effectiveness of CAZ-AVI and M/V in the treatment of infections caused by CRE [76]. The study found that the clinical success rates for both antibiotics were similar (62% for CAZ-AVI vs. 69% for M/V; *p* = 0.49), indicating no significant difference in efficacy. Notably, the use of combination therapy was higher in the CAZ-AVI group (61%) compared to the M/V group (15%; *p* < 0.01). Mortality rates after 30 and 90 days and adverse event profiles were comparable between the two groups. However, there was a concern with CAZ-AVI monotherapy, as resistance development during repeat infections was observed in three patients, an issue that was not seen in the M/V group.

### 5.3. Eravacycline

Eravacycline (ERV) is a fully synthetic fluorocycline (tetracycline class) that has been developed to treat infections caused by MDR microorganisms, such as CRE, methicillin-resistant Staphylococcus aureus, ESBL-producing *Enterobacteriaceae*, and vancomycin-resistant *enterococci* species [77,78].

Tetracycline (TET) resistance occurs through the acquisition of resistance genes which are encoded on plasmids and conjugative transposons or integrins and, therefore, can be transferred between species and genera [78]. There are currently four known mechanisms of TET resistance: efflux, ribosomal protection proteins (RPPs), ribosome mutation, and enzymatic inactivation [79]. Up to 29 genes encoding efflux pumps (e.g., tet(A) and tet(B) in Gram-negative bacteria, tet(K) in Gram-positive bacteria) and 12 genes encoding RPPs (e.g., tet(M) and tet(O) in aerobic and anaerobic bacteria) are known to cause resistance in clinically relevant pathogens [80].

ERV was designed to overcome two of the main resistance mechanisms common to the tetracycline class: ribosomal protection, commonly seen in Gram-positive organisms, and active drug efflux, common in both Gram-positive and Gram-negative organisms [77]. Like other TETs, it inhibits protein synthesis through binding to the 30S ribosomal subunit (specifically 16S rRNA).

In 2018, ERV was approved by both the EMA and the FDA for treating cIAIs [81]. In the two phase III multicenter clinical RCTs—IGNITE I and IGNITE IV—that led to its approval, ERV demonstrated non-inferiority to ertapenem and meropenem, respectively [82,83].

Despite initially also being considered a viable candidate for treating cUTIs—due to its in vitro efficacy against biofilms of uropathogenic *E. coli* [84]—ERV did not demonstrate the anticipated level of effectiveness. Two randomized, double-blind, controlled trials (NCT01978938 and NCT03032510) evaluated the safety and efficacy of intravenous ERV for cUTI (vs. levofloxacin and ertapenem) but did not demonstrate efficacy for the combined endpoints of clinical cure and microbiological success in the microbiological intent-to-treat (ITT) population [53,54].

While ERV has many attributes of an ideal antimicrobial agent—including its broad-spectrum activity—there is a need for more data on clinical efficacy and safety to fully establish its role in the treatment of infectious diseases such as cUTIs.

### 5.4. Cefiderocol

Cefiderocol, a novel synthetic siderophore-conjugated cephalosporin, was granted FDA approval in October 2019 for treating UTIs and expanded in September 2020 to address HAP and VAP. Its molecular architecture combines a cephalosporin core with a catechol-type siderophore that chelates iron, exploiting the bacterial iron transport mechanisms to gain cell entry. Upon reaching the periplasmic space, cefiderocol releases the iron, allowing its cephalosporin component to bind predominantly to PBP 3, thereby obstructing bacterial cell wall synthesis [85]. The distinctive structure and pathway of cefiderocol confer potential advantages, including resistance to the traditional mechanisms that bacteria utilize to evade antibiotics, such as the modification of porin channels, the upregulation of efflux pumps, and degradation by carbapenemases.

The approval for cefiderocol was based on the APEKS-cUTI trial, a double-blind, randomized study comparing its effectiveness to imipenem/cilastatin in adults with cUTIs [55]. The trial, encompassing several countries, recruited 448 participants and deployed a composite endpoint of clinical and microbiological response at the completion of therapy. The cefiderocol arm exhibited a superior response rate of 73% against 55% for imipenem/cilastatin, translating to an adjusted treatment difference of 18.58% (95% CI 8.23–28.92; *p* = 0.0004), thereby confirming its non-inferiority. Adverse events were recorded in 41% of cefiderocol-treated patients compared to 51% treated with imipenem/cilastatin. Microbiological efficacy was notably enhanced with cefiderocol, although clinical effectiveness was comparable between the groups. Despite its initial design to demonstrate non-inferiority, subsequent evaluations implied the superiority of cefiderocol. Notably, cefiderocol was associated with a reduced frequency of serious adverse events, with *C. difficile* colitis being the most grave.

Following the APEKS-cUTI trial, the phase III “APEKS-NP” study—a double-blind, randomized, non-inferiority trial—further evaluated cefiderocol, this time comparing it with meropenem in the treatment of hospital-acquired, community-acquired, and healthcare-associated pneumonia caused by Gram-negative pathogens [86]. This subsequent trial contributed to broadening the clinical indications for cefiderocol use.

The CREDIBLE-CR phase III trial has furthered our understanding of cefiderocol’s role in treating complex infections by comparing its efficacy with BAT in various severe infections, including cUTIs [56]. Conducted as a multicentric, randomized, open-label evaluation, the study involved patients with a spectrum of Gram-negative bacterial infections that were resistant to carbapenems. Participants were randomly assigned to receive cefiderocol or the investigator-selected BAT for 7–14 days. The results were particularly noteworthy for those with cUTIs, where cefiderocol achieved microbiological eradication in 53% of patients, compared to 20% in the BAT group. This finding suggests that cefiderocol exhibits comparable clinical and microbiological efficacy to BAT in a patient population with infections caused by carbapenem-resistant Gram-negative bacteria, positioning it as a viable treatment option, especially in the context of cUTIs where limited therapeutic alternatives exist.

A recent network meta-analysis of RCTs has identified cefiderocol as one of the leading treatment options for cUTIs, especially when considering *p*-value analysis within the subgroup of cUTI infections [87]. This is further supported by recommendations from The Infectious Diseases Society of America, which has singled out cefiderocol as the treatment of choice for cUTIs caused by CRE that show resistance to both ertapenem and meropenem [88].

### 5.5. Imipenem-Cilastatin/Relabactam

Imipenem-Cilastatin-Relabactam (IMI/REL), is a combination antibiotic therapy consisting of imipenem, a carbapenem β-lactam antibacterial agant; cilastatin, a renal dehydropeptidase inhibitor that prevents antibiotic degradation in the kidney; and relabactam, a β-lactamase inhibitor [89]. The established imipenem–cilastatin combination has been fundamental in clinical use for its broad activity against Gram-negative bacteria and certain Gram-positive organisms as well as anaerobes, but its clinical efficacy has decreased in recent years due to various resistance mechanisms [48,90].

Relabactam, part of this new drug combination, is a novel β-lactamase inhibitor that enhances the efficacy of imipenem by inhibiting class A and class C β-lactamases. By inhibiting these enzymes, relabactam restores imipenem activity against some Gram-negative β-lactamase-producing bacteria, including resistant strains of *Klebsiella* and *Pseudomonas* spp. [91,92,93].

Hence, this antibiotic is specifically designed to target MDR Gram-negative bacteria, including various strains of CRE. However, it is not effective against (MBL)-producing *Enterobacterales* and carbapenem-resistant *Acinetobacter baumannii* [94].

In 2019, the FDA approved this medication to treat cUTIs and cIAIs [95].

Clinical trials demonstrated its efficacy and safety, showing it to be comparable to or non-inferior to existing treatments, with a lower incidence of nephrotoxicity, making it a valuable addition to the treatment options for resistant bacterial infections [57,58,96,97]. In particular, the RESTORE-IMI 1 multicenter double-blind phase III RCT (NCT02452047) compared the efficacy and safety of IMI/REL vs. colistin plus imipenem in patients with imipenem-non-susceptible bacterial infections [58]. The study examined hospitalized patients with HAP/VAP, cIAIs, or cUTIs caused by imipenem-non-susceptible (but colistin- and imipenem/relebactam-susceptible) pathogens. Patients were randomized 2:1 to 5–21 days of IMI/REL or colistin plus imipenem. The primary endpoint was favorable clinical response according to infection type in the modified microbiologic ITT population. Thirty-one patients received IMI/REL and sixteen received colistin plus imipenem. Favorable overall response was observed in 71% of IMI/REL patients and 70% colistin plus imipenem patients, and 28-day mortality resulted in 10% and 30%, respectively. Serious adverse events occurred in 10% of IMI/REL and 31% of colistin plus imipenem patients. This results support IMI/REL as a suitable treatment option for serious Gram-negative infections, including those caused by carbapenem-non-susceptible pathogens in high-risk patients.

### 5.6. Plazomicin

Plazomicin represents an innovative advancement in aminoglycoside antibiotics, approved by the FDA in 2018 [88]. This synthetically derived agent, based on the structural framework of sisomicin, exhibits potent bactericidal properties by targeting the 30S ribosomal subunit. Specifically, plazomicin binds with high affinity to the 16S rRNA within the aminoacyl-tRNA site (A-site), consequently disrupting the process of protein translation [98,99].

The in vitro efficacy of plazomicin showcases comparable minimum inhibitory concentration (MIC) ranges against a spectrum of Gram-negative and Gram-positive pathogens, aligning closely with the activity profiles of established aminoglycosides like gentamicin, tobramycin, and amikacin. Plazomicin shares with other aminoglycosides a reduced effectiveness against anaerobic bacteria, both Gram-negative and Gram-positive. The broad-spectrum capability of plazomicin extends to various clinically significant bacteria, including *Enterobacteriaceae*, *Pseudomonas aeruginosa*, and *Staphylococcus* species, with noted activity against methicillin-resistant Staphylococcus aureus (MRSA). Moreover, plazomicin demonstrates a remarkable potency against pathogens resistant to conventional treatments, such as those producing ESBLs, CRE, and bacteria harboring aminoglycoside-modifying enzyme (AME) genes. These attributes position plazomicin as a crucial therapeutic option in the escalating battle against antibiotic-resistant infections. Several studies showed plazomicin to be effective in the treatment of cUTIs and pyelonephritis and have demonstrated activity against emerging clinical drug-resistant bacteria such as CRE [100].

In 2019, the Evaluating Plazomicin in Complicated Urinary Tract Infection (EPIC) trial was pivotal in establishing plazomicin’s role in treating cUTIs, underscoring the urgent need for new treatments against the rising tide of MDR Gram-negative uropathogens [59]. The trial enrolled 609 patients with cUTIs, randomizing them in a 1:1 ratio to receive either intravenous plazomicin or meropenem. The primary aim was to demonstrate plazomicin’s non-inferiority to meropenem. The trial’s results confirmed plazomicin’s comparable efficacy to meropenem, with an 88.0% success rate (clinical cure and microbiological eradication) in the plazomicin group versus 91.4% in the meropenem group. At the TOC visit, success rates were 81.7% for plazomicin and 70.1% for meropenem. Notably, plazomicin showed superior microbiological eradication, especially against aminoglycoside-resistant *Enterobacteriaceae* (78.8% vs. 68.6%) and extended-spectrum β-lactamase-producing strains (82.4% vs. 75.0%).

Furthermore, a separate phase II study compared plazomicin with levofloxacin in cUTI treatment, revealing microbiological eradication rates of 50.0%, 60.8%, and 58.6% for plazomicin at 10 or 15 mg/kg and levofloxacin at 750 mg, respectively, in the modified ITT populations [60]. The microbiologically evaluable population had eradication rates of 85.7%, 88.6%, and 81.0%, respectively, with clinical cure rates of 66.7%, 70.6%, and 65.5% in the respective groups.

Lastly, the CARE trial, a multicenter, randomized, open-label study, lent further support to plazomicin’s efficacy in serious CRE infections [101]. Comparing plazomicin-based regimens to colistin-based regimens, the study observed a numerical decrease in mortality or severe disease-related complications (23.5% vs. 50%, respectively; 90% CI −0.7 to 51.2). A preliminary analysis also indicated a lower 28-day mortality rate in the plazomicin cohort (7.1% [1/14] vs. 40.0% [6/15]). These findings, while promising, should be cautiously interpreted due to the limited sample size.

### 5.7. Aztreonam/Avibactam

Aztreonam is a monobactam antibiotic approved by the FDA in 1986 to treat various infectious diseases, including UTIs. Its strength consists of its resistance to hydrolysis by MBLs. However, monobactams are degraded by other β-lactamases that are frequently co-produced with MBLs, limiting the clinical usefulness of aztreonam monotherapy.

Avibactam is a non-β-lactam β-lactamase inhibitor that effectively inhibits serine carbapenemases. The combination of aztreonam and avibactam (ATM-AVI) is under clinical development as a response to the growing problem of infections caused by Gram-negative bacteria, including MBL-producing multidrug-resistant bacteria [102,103].

In a multicenter study involving 69 medical centers in 36 countries, the authors evaluated the in vitro activity of ATM-AVI against a global collection of CRE, including ceftazidime/avibactam-resistant isolates. In this study, ATM-AVI inhibited 99.6% of CREs at ≤8 mg/L, including 98.9% of ceftazidime/avibactam-resistant isolates [104].

This new combination drug has received the Qualified Infectious Disease Product (QIDP) and Fast Track designations from the FDA for the treatment of cIAIs, cUTIs, and HAP/VAP. These designations are intended to expedite the development and review process for drugs that treat serious conditions and fill an unmet medical need. However, ATM-AVI has not yet been formally approved by the FDA and is currently pending Phase III clinical trials (NCT03329092 and NCT03580044) [61,62]. ATM-AVI has recently shown a safety profile similar to aztreonam alone, suggesting that the addition of avibactam does not introduce new safety concerns but does enhance the antibiotic activity against resistant bacteria [105].

Despite the absence of FDA approval, clinicians can administer this combination by using two FDA-approved drugs: aztreonam and ceftazidime–avibactam. This combination of drugs is recommended by multiple experts for the treatment of serious infections caused by MBL-producing CRE [106]. This combined use reflects the clinical need for effective treatments against resistant infections and demonstrates the healthcare community’s adaptability in leveraging existing medications to address emerging challenges in infectious disease management.

## 6. Conclusions

This review has scrutinized the multifaceted challenges posed by UTIs, particularly those complicated by carbapenem-resistant *Enterobacteriaceae* (CRE). While UTIs are a common affliction globally, the advent of CRE has dramatically complicated their management, leading to an urgent call for innovative therapies.

The exploration of novel therapeutic options, as highlighted in this review, offers promising avenues to address this rising threat.

However, history’s cautionary tales of resistance development underpin the need for judicious application of these new therapies.

Overreliance on any single class of antibiotics without considering stewardship and resistance trends could inadvertently fuel the emergence of new resistance mechanisms.

As we navigate this era of considerable antimicrobial resistance, our collective actions must be informed by both current evidence and an awareness of the dynamic interplay between drug development and bacterial adaptation.

The ultimate goal remains clear: to maintain a robust arsenal against UTIs in both hospital and community settings while safeguarding the future of antibiotic therapy.

## Figures and Tables

**Table 1:** Principal characteristics of RCTs assessing novel therapeutic strategies for urinary tract infections.

Study	Trials Number	Study Design	Inclusion Criteria	Intervention Drug	Comparator Drug	Primary Outcomes
Vazquez et al., 2012 [50]	NCT00690378	Phase II, Prospective, Double-Blind, Randomized	Acute pyelonephritis or cUTI due to Gram-negativity	Ceftazidime /avibactam (n = 46)	Imipenem + cilastatin (n = 49)	Microbiological response at TOC visit
Wagenlehner et al., 2016 [51]	NCT01595438 NCT01599806	Phase III, Randomized, Double-Blind, Double-Dummy, Parallel-Group	cUTI or acute pyelonephritis requiring hospitalization	Ceftazidime /avibactam (n = 393)	Doripenem (n = 417)	Symptomatic resolution and microbiological eradication at TOC
Kaye et al., 2018 [52]	NCT02166476	Phase III, Randomized, Double-Blind, Active-Control, Double-Dummy	cUTI or acute pyelonephritis	Meropenem /vaborbactam (n = 272)	Piperacillin /tazobactam (n = 273)	Symptomatic resolution and microbial eradication at TOC
2015 [53]	NCT01978938 *	Phase III, Randomized, Double-Blind, Double-Dummy, Multicenter, Prospective	cUTI or acute pyelonephritis	Eravacycline (n = 455)	Levofloxacin (n = 453)	Non-inferiority in responder outcome in the micro-ITT population
2018 [54]	NCT03032510 *	Phase III, Randomized, Double-Blind, Double-Dummy, Multicenter, Prospective	cUTI or acute pyelonephritis	Eravacycline (n = 603)	Ertapenem (n = 602)	Non-inferiority in responder outcome in the micro-ITT population
Portsmouth et al., 2018 [55]	NCT02321800	Phase II, Double-Blind, Parallel-Group, Non-Inferiority	cUTI or acute pyelonephritis	Cefiderocol (n = 252)	Imipenem + cilastatin (n = 119)	Clinical and microbiological outcomes at TOC for non-inferiority
Bassetti et al., 2020 [56]	NCT02714595	Phase III, Randomized, Open-Label, Parallel-Group, Descriptive	Nosocomial pneumonia, sepsis, or cUTI, with carbapenem resistance and Gram-negativity	Cefiderocol (n = 101)	Best available therapy (n = 49)	Microbiological eradication at TOC in carbapenem-resistant microbiological ITT population
Sims et al., 2017 [57]	NCT01505634	Phase II, Prospective, Randomized, Double-Blind, Dose-Ranging	cUTI or acute pyelonephritis requiring hospitalization	Imipenem + cilastatin /Relebactam 250 mg (n = 99) or 125 mg (n = 99)	Imipenem/cilastatin (n = 100)	Favorable microbiological response in ME population
Motsch et al., 2020 [58]	NCT02452047	Phase III, Randomized, Double-Blind, Active Comparator, Controlled	HABP, VABP, cIAI, or cUTI by imipenem-non-susceptible pathogens	Imipenem + cilastatin /Relebactam (n = 31)	Colistimethate Sodium + Imipenem + Cilastatin (n = 16)	Favorable overall response
Wagenlehner et al., 2019 [59]	NCT02486627	Phase III, Randomized, Double-Blind	cUTI, including acute pyelonephritis	Plazomicin (n = 191)	Meropenem (n = 197)	Composite cure at day 5 and at TOC visit in micro-ITT population
Connolly et al., 2018 [60]	NCT01096849	Phase II, Double-Blind, Randomized, Controlled	cUTI or acute pyelonephritis	Plazomicin 10 or 15 mg/kg (n = 63)	Levofloxacin (n = 29)	Microbiological eradication at TOC in MITT and ME populations
2023 [61]	NCT03329092	Phase III, Prospective, Randomized, Multicenter, Open Label, Central Assessor Blinded, Comparative	Confirmed HAP/VAP or cIAI requiring IV antibiotics	Aztreonam -Avibactam ± Metronidazole (n = 282)	Meropenem ± Colistin (n = 140)	Efficacy, safety, and tolerability
2023 [62]	NCT03580044	Prospective, Randomized, Multicenter, Open-Label, Comparative	Serious bacterial infection (including cUTI) with MBL-positive Gram-negative bacteria	Aztreonam -Avibactam (n = 12)	Best available therapy (n = 3)	Proportion of subjects with clinical cure in microbiological ITT analysis

cUTI = complicated urinary tract infection; micro-ITT = microbiological intent-to-treat; TOC = test-of-cure visit; HABP = hospital-acquired bacterial pneumonia; VABP = ventilator-associated bacterial pneumonia; cIAI = complicated intra-abdominal infection; MITT: modified intent-to-treat; ME = microbiologically evaluable. * This RCT did not reach its primary endpoint.

**Table 2:** Emerging therapies for treating carbapenemase-resistant pathogens: applications and mechanisms.

Antibiotic	Drug Class	Target; Mechanism of Action	Formulation	Activity
Ceftazidime –Avibactam	Cephalosporin and DBO BLI	PBP/β-lactamase enzyme; Cell wall synthesis inhibition	2000 mg/500 mg	cUTI
Meropenem –Vaborbactam	Carbapenem and cyclic boronic acid BLI	PBP/β-lactamase enzyme; Cell wall synthesis inhibition	1 g/1 g	cUTI VAP HAP CRBSI
Eravacycline	Tetracycline	30S ribosomal subunit; Protein synthesis inhibition	50 mg	cIAI
Cefiderocol	Siderophore-β-lactam (Cephalosporin)	PBP; Cell wall synthesis inhibition	1 g	cUTI MBL
Imipenem + Cilistatin/Relebactam	Carbapenem and DBO BLI	PBP/β-lactamase enzyme; Cell wall synthesis inhibition	500 mg/500 mg/250 mg	cUTI HAP cIAI
Plazomicin	Aminoglycoside	30S ribosomal subunit; Protein synthesis inhibition	500 mg/10 mL	cUTI HAP VAP cIAI
Aztreonam –Avibactam	Monocyclic-β-lactam and DBO BLI	PBP/β-lactamase enzyme; Cell wall synthesis inhibition	500/167 mg 1500/500 mg	ESBLs and MBLs

BLI = Β-lactamase inhibitor; cIAI = complicated intra-abdominal infection; CRBSI = catheter-related bloodstream infection; cUTI = complicated urinary tract infection; DBO = diazabicyclooctane; ESBLs = extended-spectrum Β-lactamases; HAP = hospital-acquired pneumonia; MBL = metallo-Β-lactamase strain; VAP = ventilator-assisted bacterial pneumonia.

## Data Availability

All data derives from other studies. No original data are available from the corresponding author.
